# Duration of Antibody Responses after Severe Acute Respiratory Syndrome

**DOI:** 10.3201/eid1310.070576

**Published:** 2007-10

**Authors:** Li-Ping Wu, Nai-Chang Wang, Yi-Hua Chang, Xiang-Yi Tian, Dan-Yu Na, Li-Yuan Zhang, Lei Zheng, Tao Lan, Lin-Fa Wang, Guo-Dong Liang

**Affiliations:** * Shanxi Provincial Center for Disease Control and Prevention, Taiyuan, People’s Republic of China; † Shanxi Provincial Peoples’ Hospital, Taiyuan, People’s Republic of China; ‡ CSIRO Australian Animal Health Laboratory and Australian Biosecurity Center for Emerging Infectious Diseases, Geelong, Victoria, Australia; § State Key Laboratory for Infectious Disease Control and Prevention, Beijing, People’s Republic of China

**Keywords:** SARS, convalescent, antibodies, longitudinal study, dispatch

## Abstract

Among 176 patients who had had severe acute respiratory syndrome (SARS), SARS-specific antibodies were maintained for an average of 2 years, and significant reduction of immunoglobulin G–positive percentage and titers occurred in the third year. Thus, SARS patients might be susceptible to reinfection >3 years after initial exposure.

Severe acute respiratory syndrome (SARS) represents the first pandemic transmissible disease to emerge in this century. It was caused by a previously unknown coronavirus, the SARS-associated coronavirus (SARS-CoV) (*1*). SARS-CoV spreads from animals to humans by a rapid adaptation and evolution process (*2*,*3*). A large number of closely related viruses are present in wildlife reservoir populations (*4*–*6*). Therefore, due to cross-species transmission of the same or a similar coronavirus, SARS could recur. Immune protection against infection with other human coronaviruses, such as OC43 and 229E, is short-lived (*7*). To assess SARS patients’ risk for future reinfection, we conducted a longitudinal study of immunity in convalescent patients.

## The Study

Shanxi Province in China was 1 of the SARS epicenters during the 2002–03 outbreaks. For our study, serum samples were taken from patients in 7 designated SARS hospitals in the province during March–August 2003. Follow-up serum samples were taken at 6 months, 1, 2, and 3 years after the onset of symptoms. A total of 176 cases that met the World Health Organization (WHO) SARS case definition (*8*) and had known transmission history were included in this study. The study was conducted as part of a national SARS control and prevention program; use of serum from human participants was approved by the Committee for SARS Control and Prevention, Department of Science and Technology, the People’s Republic of China.

Titers of serum antibodies to SARS-CoV were determined by using a commercially available ELISA kit (BJI-GBI Biotechnology, Beijing, China). The ELISA was based on an inactivated preparation of whole-virus lysate. The kit was the first commercial kit approved by the Chinese Food and Drug Administration for specific detection of SARS-CoV antibodies and has been widely used in several studies (*9*–*11*). Manufacturer’s instructions were followed without modification. Briefly, for every ELISA plate, 1 blank, 1 positive, and 2 negative controls were included. For detection of immunoglobulin G (IgG), a 1:10 dilution of testing serum (100 μL) was added to antigen-coated wells, and the plate was incubated at 37^o^C for 30 min. Horseradish peroxide (HRP)–conjugated antihuman IgG (100 μL) was then added for detection of bound antibodies. For detection of IgM, the incubation of primary antibodies was extended to 60 min, followed by detection with HRP-conjugated antihuman IgM. Optical density (OD) readings were deemed valid only when the negative control OD was <0.10 and the positive control was >0.50 on the same ELISA plate. The cutoff for IgG and IgM determination was defined as 0.13 and 0.11, respectively, plus OD of the negative control. When the OD of the negative control was <0.05, 0.05 was used for the calculation. In this study, the OD readings of negative controls from different testing were consistently <0.05, so the cutoff ODs for IgG and IgM were 0.18 and 0.16, respectively. Serum samples that had an OD greater than or equal to the cutoff value were considered positive. Weak positive samples (i.e., OD<2× cutoff value) were retested in duplicate on the same day; only reproducible positive results were included in the final analysis. All data were processed by using Excel version 7.0 (Microsoft Corp., Redmond, WA, USA) and SPSS software version 10 for Windows (SPSS Inc., Chicago, IL, USA).

**Figure 1:**
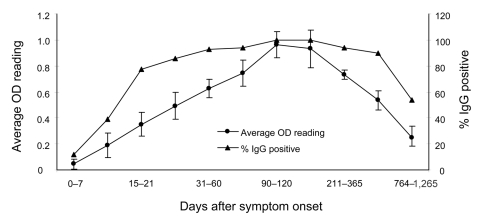
Change of immunoglobulin G (IgG) patterns among 176 convalescent severe acute respiratory syndrome patients with known transmission history. See the Table for number of samples used for the calculation at each time point. OD, optical density.

Among the cohort, 163 (92.61%) of 176 (χ^2^ = 200.11, p = 0.000002) were IgG positive, which indicated that most patients who met the WHO case definition were indeed infected with SARS-CoV. As shown in the Table, at ≈7 days after the onset of symptoms, the percentage who were IgG positive was ≈11.80%. This percentage continued to increase, reached 100% at 90 days, and remained largely unchanged up to 200 days. Furthermore, after 1 and 2 years 93.88% and 89.58% of patients, respectively, were IgG positive, which suggests that the immune responses were maintained in >90% of patients for 2 years. However, 3 years later, ≈50% of the convalescent population had no SARS-CoV–specific IgG. The OD changes correlated with the changes to the IgG-positive percentage, although the rate of change varied. Both the OD readings (0.93) and positive percentages peaked at 90–120 days; however, the rate of reduction of the average OD readings was much faster, dropping by 22% (0.73) and 40% (0.54) at 1 and 2 years, respectively, after symptom onset (Figure 1).

A similar observation was obtained for IgM trends in this same cohort. The percentage of patients who were IgM positive within the first 7 days was 21.4% and peaked at 76.2% after 21–30 days (Table). The patterns of IgM-positive percentage and average OD readings were similar; both peaked at 21–30 days. After 60 days, the average OD readings dropped to 0.167, close to the cutoff value of 0.160.

**Table:** Cumulative rates of SARS-CoV antibodies among 176 SARS patients with known transmission histories*

Time after symptom onset, d	IgG				IgM†		
	No. samples tested	No. positive samples (%)	Average OD		No. samples tested	No. positive samples (%)	Average OD
0–7	17	2 (11.76)	0.046		14	3 (21.43)	0.136
8–14	26	10 (38.46)	0.190		22	14 (63.64)	0.312
15–20	22	17 (77.27)	0.351		19	12 (63.16)	0.477
21–30	36	33 (91.67)	0.493		21	16 (76.19)	0.560
31–60	72	67 (93.06)	0.627		22	14 (63.64)	0.320
61–90	35	33 (94.29)	0.745		15	5 (33.33)	0.167
91–120	11	11 (100.00)	0.965		ND	ND	ND
121–210	23	23 (100.00)	0.932		ND	ND	ND
211–365	49	46 (93.88)	0.734		ND	ND	ND
366–763	96	86 (89.58)	0.535		ND	ND	ND
764–1,265	28	15 (53.57)	0.250		ND	ND	ND

*SARS-CoV, severe acute respiratory syndrome–associated coronavirus; Ig, immunoglobulin; OD, optical density; ND, not determined because for most samples the IgM readings already reached background level on day 90. †For some patients, we did not have enough serum to test for IgM after testing for IgG; hence, a smaller number of serum samples were tested for IgM than for IgG.

**Figure 2:**
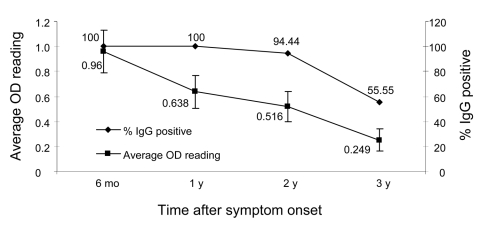
Change of immunoglobulin G (IgG) patterns among 18 convalescent severe acute respiratory syndrome patients with a complete collection of sequential serum samples at the time points shown. The 18 patients were selected from the cohort of 176 patients for whom transmission history was known. OD, optical density.

Among the cohort of patients with known transmission histories, we were able to obtain a complete collection of serum samples from 18 patients at 6 months, 1, 2, and 3 years. The IgG levels of these 18 patients were analyzed separately to obtain an IgG trend that more accurately represented convalescent SARS patients (Figure 2). All 18 patients had positive IgG at 6 months and at 1 year (i.e., 100% positive); only 1 patient became IgG negative at 2 years. However, at 3 years, the positive percentage dropped to 55.56%. The reduction of OD values mimicked that of the positive percentage, again at a faster rate. The average OD readings dropped from 0.94 at 6 months to 0.64 at 1 year, which represents a reduction of 33.33%. The OD further dropped to 0.52 (45.83% reduction) by 2 years and to 0.25 by 3 years.

## Conclusions

To our knowledge, the 3-year follow-up conducted in this study is the longest longitudinal study ever reported. With a large number of patients who had confirmed transmission history (176) and a complete dataset for 18, the level of confidence is high that the results obtained in this study are representative for convalescent SARS patients. Similar results have been reported from longitudinal studies of SARS patients with smaller cohort size (18–98 patients) and shorter follow-up period (240 days to 2 years) (*9*–*14*). The general trend of IgM peaking at ≈1 month after symptom onset and IgG peaking at 2–4 months was consistent among different studies.

Our results provide strong evidence that SARS-CoV antibodies are reduced >3 years after the symptom onset. Because antibodies play an important role in protective immunity against SARS-CoV (*15*), the findings from this study will have important implications with regard to assessing risk for reinfection among previously exposed populations (e.g., hospital staff) and evaluating the duration of antibody-mediated immunity that any candidate vaccine could provide.
